# Acetate-free dialysate, protein-energy wasting, and muscle outcomes in maintenance hemodialysis: a randomized-controlled trial

**DOI:** 10.1093/ckj/sfag323

**Published:** 2026-09-22

**Authors:** Julien Aniort, Solene Grall, Valerie Batel, Bruno Pereira, Anne-Elisabeth Heng

**Affiliations:** Department of Nephrology, Dialysis and Transplantation, Gabriel Montpied Hospital, CHU Clermont-Ferrand, Clermont-Ferrand, France; Human Nutrition Unit, INRAE UMR 1019, Clermont Auvergne University, Clermont-Ferrand, France; Department of Nephrology, Dialysis and Transplantation, Gabriel Montpied Hospital, CHU Clermont-Ferrand, Clermont-Ferrand, France; Department of Nephrology, Dialysis and Transplantation, Gabriel Montpied Hospital, CHU Clermont-Ferrand, Clermont-Ferrand, France; Biostatistics Unit, CHU Clermont-Ferrand, Clermont-Ferrand, France; Department of Nephrology, Dialysis and Transplantation, Gabriel Montpied Hospital, CHU Clermont-Ferrand, Clermont-Ferrand, France; Human Nutrition Unit, INRAE UMR 1019, Clermont Auvergne University, Clermont-Ferrand, France

**Keywords:** acetate, dialysate, hemodialysis, muscle, protein energy wasting

## Abstract

**Background:**

Protein–energy wasting and muscle atrophy are common in maintenance hemodialysis and linked to adverse outcomes. Acetate from acetic acid–containing dialysate concentrate may promote inflammation and metabolic disturbances. We evaluated whether acetate-free dialysate prepared from a hydrochloric acid–containing concentrate could improve nutritional status and muscle-related outcomes in chronic hemodialysis patients.

**Methods:**

In this single-center, single-blind, randomized crossover trial, adults receiving maintenance hemodialysis were assigned to acetate-free or acetate-containing dialysate for 6 months, followed by crossover to the alternate dialysate for an additional 6 months. The primary endpoint was serum prealbumin. Secondary endpoints included body composition assessed by bioimpedance spectroscopy, handgrip strength, 4-m gait speed, SARC-F score, serum albumin, and C-reactive protein. Outcomes were analyzed using linear mixed-effects models adjusted for period and treatment sequence, with patient included as a random effect.

**Results:**

Sixty patients were included. The mean age was 68.8 ± 9.4 years, and 72.4% were male. Prealbumin concentrations remained stable with acetate-containing dialysate, from 0.291 ± 0.079 g/l at baseline to 0.287 ± 0.076 g/l at month 6, and with acetate-free dialysate, from 0.294 ± 0.085 to 0.279 ± 0.074 g/l. The dialysate-by-time interaction was not significant (*P* = .386). Lean tissue index, fat tissue index, handgrip strength, 4-m gait speed, serum albumin, and C-reactive protein remained unchanged. Safety outcomes, including infectious and cardiovascular events and mortality, did not differ significantly between strategies.

**Conclusion:**

In this randomized crossover trial, acetate-free dialysate did not improve nutritional and muscle status compared with conventional acetate-containing dialysate.

## Graphical Abstract

**Graphical Abstract:**
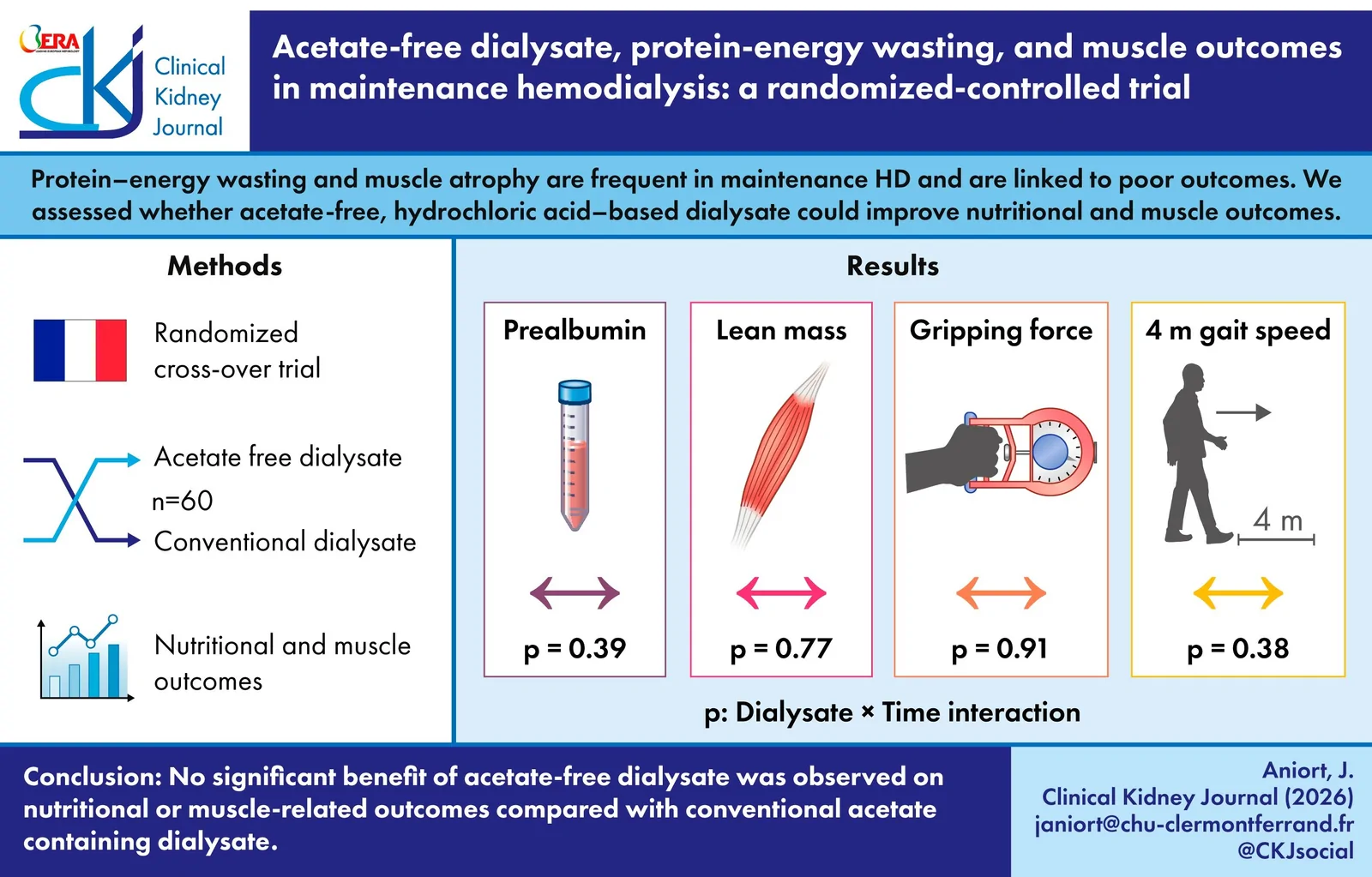

KEY LEARNING POINTS **What was known:**

- Protein–energy wasting and sarcopenia are common in chronic hemodialysis patients and are associated with mortality, functional decline, and impaired quality of life.
- Low-grade inflammation, driven partly by dialysis-related bioincompatibility, contributes to muscle wasting, and nutritional deterioration in hemodialysis patients.
- Acetate-containing dialysate may promote inflammation, oxidative stress and hemodynamic instability, but the long-term nutritional effects of acetate-free hydrochloric-acid-based dialysate is unknown.

**This study adds:**

- In this randomized crossover trial, acetate-free dialysate did not improve serum prealbumin compared with conventional low-acetate dialysate.
- Acetate-free dialysate did not favorably affect lean tissue index, fat tissue index, handgrip strength, gait speed, SARC-F score, albumin, or C-reactive protein.
- Acetate-free dialysate was well tolerated and showed a safety profile comparable to conventional acetate-containing dialysate, with no significant difference in mortality.

**Potential impact:**

- In contemporary hemodialysis practice with low acetate exposure and adequate metabolic acidosis control, acetate removal alone may not provide clinically meaningful nutritional benefit.
- Routine use of hydrochloric-acid-based acetate-free dialysate cannot be recommended solely to improve protein–energy wasting or sarcopenia in chronic hemodialysis patients.
- Larger randomized trials are needed to determine whether acetate-free dialysate may influence harder clinical outcomes, including survival, in selected hemodialysis populations.

## INTRODUCTION

Chronic hemodialysis patients are at high risk of protein–energy wasting (PEW). It is defined as the presence of at least one criterion in three of four categories: altered biochemical nutritional markers, reduced body weight or fat mass, decreased muscle mass, and insufficient dietary intake [1]. PEW is associated with increased mortality and poorer quality of life in chronic hemodialysis patients [2]. Muscle atrophy is a key component of PEW, independently associated with mortality and leading to reduced autonomy and impaired quality of life [3]. Despite its clinical importance, the effectiveness of current interventions, including nutritional supplementation and physical exercise, remains limited. Low-grade inflammation promotes PEW and muscle atrophy by enhancing muscle protein degradation through activation of the ubiquitin–proteasome pathway and the autophagy–lysosome system [4]. It is partly driven by dialysis-related bioincompatibility, involving the dialysis membrane, extracorporeal circuit, and dialysate [5].

Dialysate is produced from an acidic, calcium-containing concentrate and a bicarbonate-containing concentrate. Their reaction generates carbon dioxide, maintaining an acidic pH and preventing calcium carbonate precipitation [6]. Acetic acid is typically used in acidic concentrates and reacts with bicarbonate to form acetate, resulting in dialysate acetate concentrations of ∼3 mmol/l. This level, nearly 20 times higher than physiological plasma acetate concentrations, leads to acetate transfer into the circulation. Elevated plasma acetate has been associated with inflammation and hemodynamic instability. Acetate stimulates the production of proinflammatory cytokines [7], increases oxidative stress [8], and promotes arterial hypotension via induction of nitric oxide synthase [9]. Because chronic inflammation contributes substantially to PEW and muscle atrophy, reducing acetate exposure may help preserve muscle mass and nutritional status.

One possible alternative to acetic acid is its replacement with hydrochloric acid. Like acetic acid, hydrochloric acid reacts with part of the bicarbonate to generate carbon dioxide and water. However, because hydrochloric acid is a strong acid, it leaves chloride rather than acetate as the conjugate base, thereby producing an acetate-free dialysate. However, this approach increases bicarbonate consumption and requires adaptations in the use of dialysis machines. The long-term effects of an acetate-free dialysate based on hydrochloric acid concentrate on PEW and sarcopenia have not yet been investigated. The objective of this study was therefore to evaluate whether this acetate-free dialysate reduces PEW in chronic hemodialysis patients compared with conventional acetate-containing dialysate.

## MATERIALS AND METHODS

### Study design

A single-center, single-blind, randomized, crossover clinical trial comparing acetate-containing (A + D) and acetate-free dialysate (A − D) was conducted at the hemodialysis center of Clermont-Ferrand University Hospital between March 2021 and March 2025. Patients were randomly assigned in a 1:1 ratio to one of two treatment sequences using a computer-generated randomization sequence. Patients in the first group received A − D for 6 months, followed by A + D for the subsequent 6 months. Patients in the second group continued their usual A + D for 6 months and then switched to A − D for the following 6 months. Total follow-up duration was 12 months. No washout period was implemented, as interruption of dialysis was not feasible (Fig. 1). Participants and outcome assessors were blinded to treatment allocation. The dialysate concentrate container was concealed from participants after connection to the dialysis machine. Dialysis staff were not blinded.

**Figure 1:**
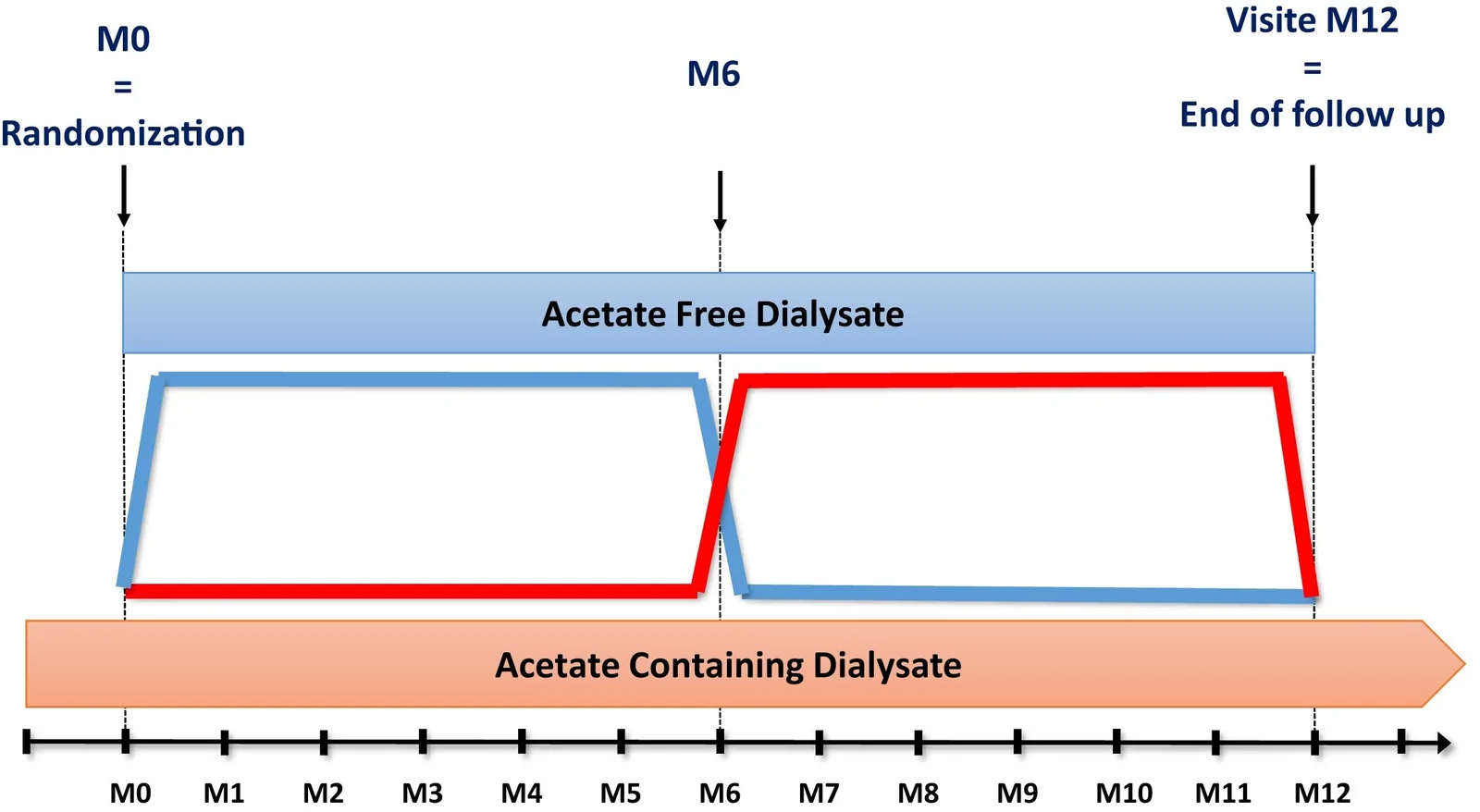
Study design. A − D acetate free dialysate A + D acetate containing dialysate.

### Study population

Eligible participants were adult patients (>18 years) with end-stage kidney disease undergoing chronic intermittent hemodialysis for at least 6 months using A + D. Exclusion criteria were: lower limb amputation proximal to a transmetatarsal amputation, an acute event within the 3 months prior to inclusion (infection or surgery), neuromuscular disorders, an estimated life expectancy of less than 6 months, psychiatric disorders or cognitive impairment precluding informed consent, pregnancy, and legal protection status (guardianship or curatorship). The study was approved by the French Research Ethics Committee Sud-Est VI (approval number 2020-A00678-31) and registered on ClinicalTrials.gov (identifier NCT04824885). All participants were affiliated with a social security scheme, provided written informed consent after receiving detailed information about the study protocol, and were considered able to comply with study procedures.

### Interventions

Hemodialysis or post-dilution online hemodiafiltration sessions were performed using Fresenius 6008 (Fresenius Medical Care, Bad Homburg, Germany) or DBB-EXA dialysis machines (Nikkiso Co., Ltd, Tokyo, Japan). Treatments were delivered three times per week, with a session duration of 4–5 h. High-flux polysulfone membranes were used: ELISIO 21H (Nipro Corporation, Osaka, Japan) for hemodiafiltration and Dialhemo 22 (Medtronic, Minneapolis, MN, USA) for conventional hemodialysis. The prescribed blood flow rate ranged from 300 to 400 ml/min, and the dialysate flow rate was 500 ml/min. Prescribed dry weight was left to the discretion of the attending nephrologist. Anticoagulation of the extracorporeal circuit was achieved using unfractionated heparin. During the A + D period, the acid concentrate used was NIPRO BAG 300A (Nipro Corporation, Osaka, Japan) or SOFTPACK Series (Fresenius Medical Care, Bad Homburg, Germany), both at a dilution of 1:45. During the A-D period, the acid concentrate was LYMPHA (Medtronic, Minneapolis, MN, USA), also at a dilution of 1:45. The composition of the dialysates is detailed in Table 1. Dialysate sodium concentration was set according to the treating nephrologist’s prescription. Bicarbonate concentration was set at 32 mmol/l A + D and 34 mmol/l with A − D to achieve an equivalent bicarbonate load.

**Table 1:** Composition of the acid concentrates used in the study.

	Acid concentrate composition		Final dialysate concentration (mmol/l)	
Acetate containing dialysate (A + D)	NaCl	270,88 g	Sodium	140
	CaCl_2_,2H_2_O	9.92 g	Potassium	2
	MgCl_2_,6H_2_O	4.57 g	Chloride	109
	KCL	6.71 g	Calcium	1.5
	CH_3_COOH	8.11 g	Magnesium	0.5
	C_6_H_12_O_6_,H_2_O	49.50 g	Bicarbonate	34
	H_2_O Ph/Eur. Q.s.	1000 mL	Acetate	3
			Dissolved CO_2_	3
			Glucose	5.5
Acetate free dialysate (A − D)	NaCl	270,88 g	Sodium	140
	CaCl_2_, 2H_2_O	9.92 g	Potassium	2
	MgCl_2_, 6H_2_O	4.57 g	Chloride	112
	KCL	6.71 g	Calcium	1.5
	HCl	4.92 g	Magnesium	0.5
	C_6_H_12_O_6_	45 g	Bicarbonate	34
	H_2_O Ph/Eur. Q.s.	1000 ml	Acetate	0
			Dissolved CO_2_	2
			Glucose	5.5

The data are those provided by the manufacturers. Each concentrate is available with a final potassium concentration of 2 or 3 mmol/l.

### Endpoints

The primary endpoint was serum prealbumin concentration (g/l), measured by latex immunoturbidimetry (Atellica®, Siemens). Prealbumin is a validated marker included in the International Society of Renal Nutrition and Metabolism criteria for PEW [1]. Its short half-life allows it to respond more rapidly than albumin or measures of muscle mass to changes in catabolic status. Secondary endpoints included body weight (kg) and body composition parameters assessed by bioelectrical impedance spectroscopy using the Body Composition Monitor (BCM, Fresenius Medical Care, Bad Homburg, Germany), providing measurements of total body water, extracellular and intracellular water, overhydration (OH), lean tissue mass (LTM, kg), lean tissue index (LTI, kg/m²), fat tissue mass (FTM, kg), and fat tissue index (FTI, kg/m²). Muscle strength was evaluated using handgrip dynamometry with a JAMAR® hydraulic hand dynamometer (Patterson Medical, Warrenville, IL, USA), For each participant, three measurements were performed, and both the mean and the maximal value were recorded (kg). Physical performance was assessed using the 4-m gait speed test (m/s), and sarcopenia risk was evaluated using the SARC-F questionnaire [10]. Serum albumin (g/l) was measured by immunoturbidimetry with Diagam reagent (Atellica®, Siemens), and C-reactive protein (CRP, mg/l) was measured by PEG-enhanced immunoturbidimetry (Atellica®, Siemens). Parathyroid hormone (PTH) was measured by immunochimiluminescence sandwich (Atellica®, Siemens).

### Data collection

Data were prospectively collected at predefined time points using a web-based electronic case report form (REDCap). Baseline assessments were performed at inclusion (M0), and follow-up evaluations were conducted every 6 months during routine dialysis sessions (M6 and M12). At baseline, demographic characteristics, dialysis vintage, cause of end-stage kidney disease and Charlson comorbidity index were recorded. At each visit, anthropometric measurements, including prescribed dry weight, body composition, SARC-F score, handgrip strength, and 4-m gait speed were assessed. Dialysis-related parameters were documented, including modality, session duration, dialyzer type, vascular access, blood and dialysate flow rates, and delivered dialysis dose using the Daugirdas formula [11]. Routine pre- and post-dialysis blood samples provided biochemical data, including electrolytes, mineral metabolism parameters, urea, creatinine, inflammatory and nutritional markers, and lipid profile. The normalized protein catabolic rate was calculated according to the Daugirdas formula [12]. Concomitant treatments and adverse clinical events, including death, were recorded throughout the study.

### Statistical analysis

The study was powered to detect a 0.05 g/l difference in prealbumin between A − D and A + D, assuming a standard deviation of 0.08 g/l, a two-sided α of 0.05, and 90% power. Fifty-six patients were required, and 60 were enrolled to account for potential attrition. Categorical variables are presented as counts and percentages and continuous variables as mean ± standard deviation or median [interquartile range], according to their distribution. Normality was assessed using the Shapiro–Wilk test. Primary analyses followed the intention-to-treat principle, with participants analyzed according to their randomized dialysate sequence. Per-protocol analyses excluded participants lost to follow-up or exposed to an incorrect dialysate during at least one dialysis session. Longitudinal outcomes were analyzed using linear mixed-effects models, with patient included as a random effect. First, changes over time were assessed separately for each dialysate, with adjustment for study period. Second, the comparative dialysate effect over time was assessed using the dialysate-by-time interaction, with dialysate, time, period, and treatment sequence included as fixed effects. Models were fitted by restricted maximum likelihood using all available observations under the missing-at-random assumption. Residual normality was assessed as described above. Sensitivity analyses included per-protocol and first-period-only analyses. For the primary outcome, missing 6-month data were additionally handled using multiple imputation by chained equations, with 20 imputed datasets combined according to Rubin’s rules. Survival was analyzed using a marginal Cox proportional hazards model with dialysate exposure as a time-varying covariate. Within-patient correlation was accounted for using clustered robust standard errors, and the model was adjusted for age, sex, and Charlson comorbidity index. Analyses were performed using R (v4.5.0) and Stata (version 15, StataCorp, College Station, TX, USA). Two-sided *P* values < .05 were considered statistically significant. No multiplicity adjustment was applied to secondary outcomes, which were considered exploratory and interpreted using nominal *P* values.

## RESULTS

### Patients

Between March 2021 and March 2024, 60 patients were randomized. The study flow chart is presented in Fig. 2. Two participants left the dialysis center before undergoing any post-randomization study procedure, one because of kidney transplantation and the other because of transfer to another dialysis center. During follow-up, 15 patients discontinued because of transfer, transplantation, or death, and four received the incorrect dialysate at least once. Overall, 58 patients were included in the intention-to-treat analysis and 39 in the per-protocol analysis. Available data by sequence, period, dialysate, and time point are shown in Supplementary Table S1. Follow-up of the last participant was completed in March 2025. Baseline characteristics are summarized in Table 2.

**Figure 2:**
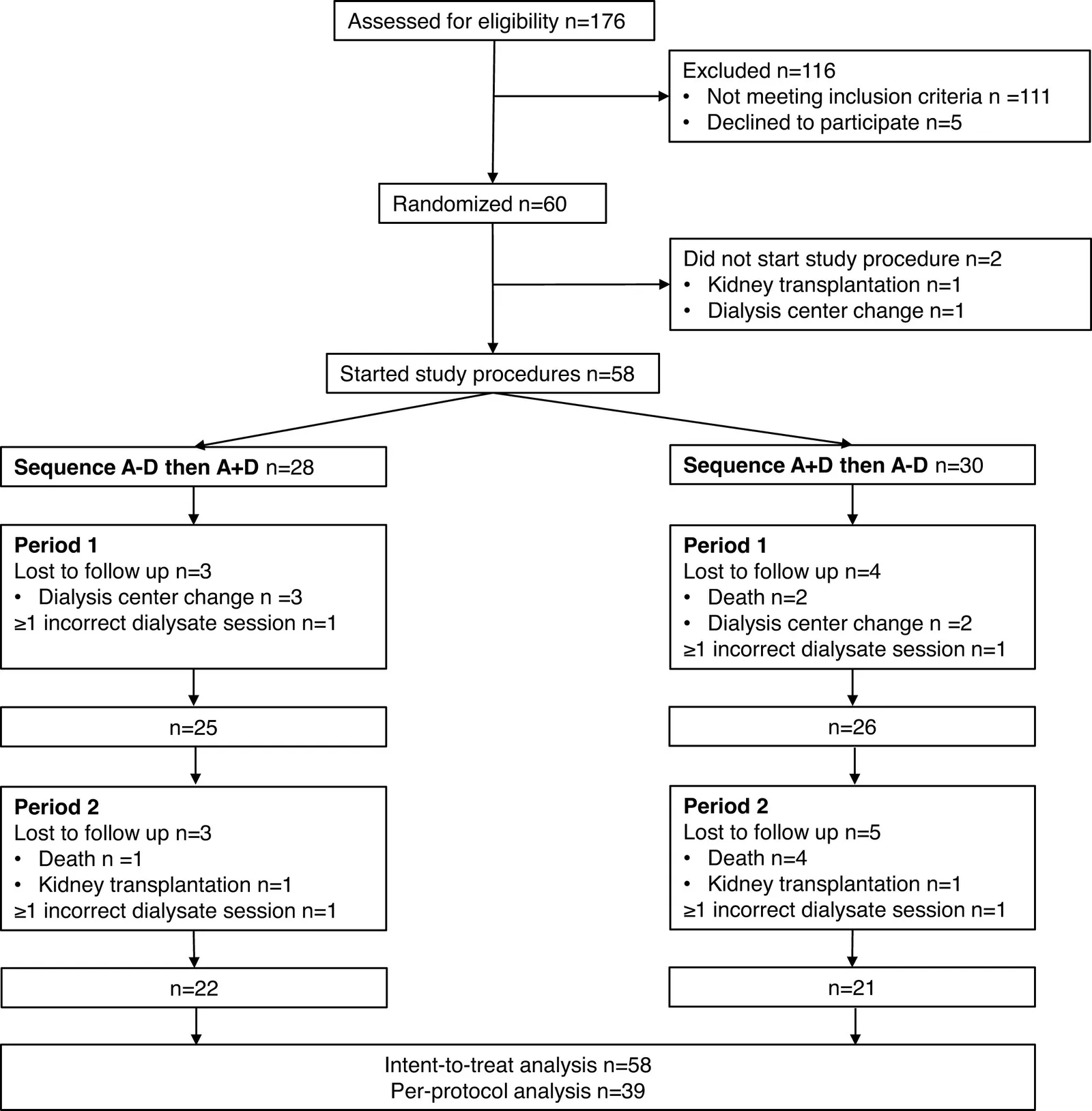
Study flow chart of patient disposition. A − D acetate free dialysate A + D acetate containing dialysate.

**Table 2:** Baseline characteristics.

Characteristics	Total (*n* = 58)	A − D then A + D (*n* = 28)	A + D then A − D (*n* = 30)
Age (years)	68.8 ± 9.4	69.2 ± 8.7	68.5 ± 10.0
Male (*n*,%)	43 (74.1)	23 (82.1)	20 (66.7)
Weight (kg)	74.7 ± 17.5	77.5 ± 11.6	72.0 ± 21.6
Height (cm)	168.3 ± 7.7	169.9 ± 7.2	166.8 ± 7.9
Charlson Comorbidity Index	3.5 ± 1.35	3.8 ± 1.45	3.3 ± 1.24
Dialysis vintage (years)	1.47 (0.38–3.65)	1.50 (0.50–3.50)	1.45 (0.33–3.50)
Cause of kidney failure, *n* (%)			
Diabete	18 (31.0)	11 (39.3)	7 (23.3)
Hypertension	13 (22.4)	6 (21.4)	7 (23.3)
Glomerulonephritis	13 (22.4)	5 (17.9)	8 (26.7)
Tubulo-interstitial nephritis	4 (6.9)	2 (7.1)	2 (6.7)
PKRD	7 (12.0)	4 (14.2)	3 (10.0)
Other	3 (5.2)	0 (0)	3 (10.0)
Biology			
Hemoglobin (g/l)	11.8 ± 1.8	11.8 ± 1.8	11.9 ± 1.8
Sodium (mmol/l)	136 ± 3.5	136 ± 2.7	136 ± 4.2
Potassium (mmol/l)	4.6 ± 0.7	4.6 ± 0.8	4.6 ± 0.7
Bicarbonate (mmol/l)	23.1 ± 2.5	23.3 ± 2.24	23.0 ± 2.7
Prealbumin (g/l)	0.303 ± 0.082	0.301 ± 0.092	0.306 ± 0.072
Albumin (g/l)	36.9 ± 4.7	36.9 ± 4.9	36.8 ± 4.5
Total cholesterol (g/l)	1.57 ± 0.46	1.58 ± 0.41	1.56 ± 0.51
LDL cholesterol (g/l)	0.78 ± 0.38	0.85 ± 0.38	0.71 ± 0.38
HDL cholesterol (g/l)	0.41 ± 0.19	0.41 ± 0.19	0.42 ± 0.18
Triglycerides (g/l)	2.17 ± 1.66	2.07 ± 1.26	2.27 ± 1.98
Urea (mmol/l)	18.8 ± 5.4	18.6 ± 5.6	18.9 ± 5.3
Creatinine (µmol/l)	642 ± 186	638 ± 188	645 ± 188
CRP (mg/l)	11.2 ± 10.5	10.6 ± 11.0	11.7 ± 10.2
nPCR (g/kg/j)	1.06 ± 0.28	1.05 ± 0.29	1.08 ± 0.29
Medication (*n*,%)			
Statin therapy	20 (34.5)	13 (46.4)	7 (23.3)
Beta blocker therapy	26 (44.8)	13 (46.4)	13 (43.3)
Erythropoietin therapy	34 (58.6)	17 (56.7)	17 (58.6)
Oral nutritional supplements	15 (25.9)	9 (32.1)	6 (20.0)
Intradialytic parenteral nutrition	2 (3.5)	1 (3.6)	1 (3.3)
Dialysis modality (*n*,%)			
Hemodialysis	41 (70.7)	18 (64.3)	23 (76.7)
Hemodiafiltration	17 (29.3)	10 (35.7)	7 (23.3)
Dialysis duration (min)	244 ± 13	242 ± 8	245 ± 16
Vascular access (*n*,%)			
AVF	48 (82.8)	24 (85.7)	24 (80.0)
Catheter	10 (17.2)	4 (14.3)	6 (20.0)
Blood flow rate (ml/min)	385 ± 25	386 ± 23	385 ± 27
Dialysate flow rate (mL/min)	500 ± 0	500 ± 0	500 ± 0
Urea Reduction Rate %	78.8 ± 0.1	78.1 ± 0.1	79.5 ± 0.1
Kt/V_sp_	1.85 ± 0.35	1.80 ± 0.31	1.90 ± 0.37
Kt/V_eq_	1.62 ± 0.30	1.56 ± 0.27	1.67 ± 0.32

Continuous variables are expressed as mean ± standard deviation or median (interquartile range). AVF, arteriovenous fistula; LDL, low density lipoprotein; HDL, high density lipoprotein; CRP, C-reactive protein; nPCR, normalized protein catabolic rate.

### Primary endpoint

In the A + D group, prealbumin was 0.291 ± 0.079 g/l at M0 and 0.287 ± 0.076 g/l at M6. In the A–D group, values changed from 0.294 ± 0.085 g/l to 0.279 ± 0.074 g/l (Fig. 3). The effect of time adjusted by period was not statistically significant in either dialysate (A + D: *P* = .72, A–D: *P* = .20). The interaction between time and dialysate type was not statistically significant (*P* = .39), indicating that prealbumin did not evolve differently over time according to dialysate type (Table 3). The analysis restricted to the first study period, yielded consistent results (Supplementary Table S3). Similarly, the sensitivity analysis performed after imputation of missing follow-up data showed no significant dialysate-by-time interaction (*P* = .45).

**Figure 3:**
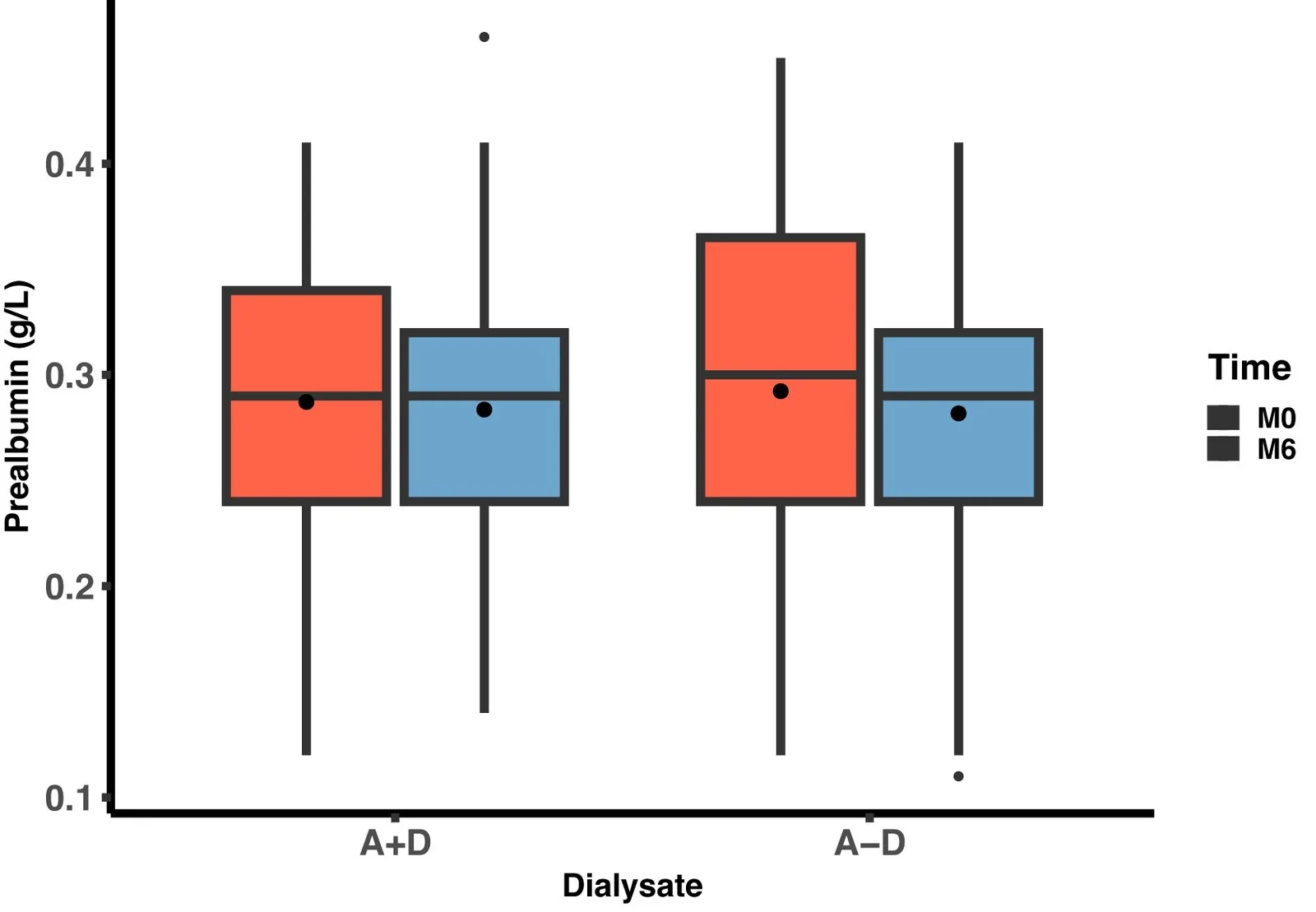
Effect of acetate-free dialysate on prealbumin levels. The figure presents box plots of prealbumin levels at baseline (M0) and at the end of the study period 6 months later (M6) according to dialysate type: acetate-free (A − D) or acetate-containing (A + D).

**Table 3:** Endpoints related to protein-energy wasting and muscle outcomes.

	A − D			A + D					
	M0 *n* = 54	M6 *n* = 46	*P*	M0 *n* = 55	M6 *n* = 48	*P*	*P* ^1^	*P* ^2^	*P* ^3^
Primary endpoint									
Prealbumin (g/l)	0.294 ± 0.085	0.279 ± 0.074	.20	0.291 ± 0.079	0.287 ± 0.076	.72	.39	.58	.15
Secondary endpoints									
Weight (kg)	75.8 ± 17.1	73.9 ± 17.8	<.01	73.9 ± 17.8	74.4 ± 17.8	.81	<.01	.30	.01
LTI (kg/m^2^)	11.7 ± 2.8	11.7 ± 2.5	.98	11.6 ± 2.6	11.5 ± 2.9	.84	.77	.79	.75
LTM (kg)	33.4 ± 9.3	33.6 ± 8.4	.99	33.1 ± 8.6	32.5 ± 9.4	.86	.78	.48	.73
FTI (kg/m^2^)	15.2 ± 7.2	14.4 ± 6.8	.32	14.6 ± 7.0	15.3 ± 7.6	.62	.15	.75	.85
FTM (kg)	31.3 ± 14.5	30.0±13.9	.39	30.1 ± 14.0	31.1 ± 15.0	.62	.17	.62	.92
OH (l)	0.88 ± 1.50	1.02 ± 1.56	.65	0.83 ± 1.67	0.50 ± 1.49	.27	.28	.81	.50
Average gripping force (kg)	25.2 ± 8.5	25.9 ± 9.1	.53	25.5 ± 8.9	25.9 ± 9.8	.62	.83	.73	.42
Maximal gripping force (kg)	26.6 ± 8.5	27.3 ± 9.4	.61	26.7 ± 9.1	27.3 ± 9.8	.65	.91	.75	.50
SARC-F score	2.1 ± 2.2	2.2 ± 2.4	.59	2.6 ± 2.3	2.4 ± 2.5	.45	.25	.16	.86
4 m gait speed (m/s)	0.89 ± 0.28	0.88 ± 0.25	.80	0.85 ± 0.29	0.91 ± 0.30	.15	.38	.68	.14
Albumin (g/l)	36.3 ± 4.0	36.1 ± 3.4	.69	36.4 ± 4.2	35.7 ± 2.8	.26	.65	.78	.24
CRP (mg/l)	11.1 ± 11.9	15.0 ± 24.6	.32	15.8 ± 21.5	11.9 ± 12.8	.24	.053	.18	.87

Continuous variables are expressed as mean ± standard deviation. A − D, acetate free dialysate; A + D, acetate containing dialysate; CRP, C-reactive protein; FTI, fat tissue index; FTM, fat tissue mass; LTI, lean tissu index; LTM, lean tissue mass; OH, overhydratation. *P: P* value for time effect adjusted for period with each dialysate. *P*^1^: *P* value for the dialysate-by-time interaction. *P*^2^: *P* value for sequence effect. *P*^3^: *P* value for period effect.

### Secondary endpoints

Detailed results for all secondary endpoints are presented in Table 3. Body weight decreased significantly over time in the A–D group (*P* < .001), whereas it remained stable in the A + D group (*P* = .81). The time-by-dialysate interaction was significant (*P* = .01), indicating a differential evolution according to dialysate type. In contrast, body composition parameters remained stable. Lean tissue index did not change significantly in either group (*P* = .77), nor did fat tissue index (*P* = .15). Overhydration levels were also unchanged over time (*P* = .28). Average and maximal handgrip strength, showed no significant longitudinal change (*P* = .83 and *P* = .91, respectively). Similarly, SARC-F score and 4-m gait speed remained stable, with no significant interaction between time and dialysate strategy (*P* = .25 and *P* = .38, respectively). Serum albumin and C-reactive protein concentrations remained unchanged, with no significant time-by-dialysate interaction (*P* = .65 and *P* = .053, respectively). Similar results were observed in per-protocol analysis (Supplementary Tables S2 and S3).

Despite a higher prescribed bicarbonate conductivity with the A − D dialysate, both pre- and post-dialysis bicarbonate concentrations decreased over time, from 22.9 ± 2.7 mmol/l at M0 to 21.7 ± 2.6 mmol/l at M6 (*P* = .01) before dialysis, and from 27.1 ± 1.6 mmol/l at M0 to 25.8 mmol/l at M6 (*P* < .001) after dialysis. The change in PTH levels differed according to dialysate type. With A + D , PTH levels remained stable, from 341 ± 293 ng/l to 346 ± 276 ng/l (*P* = .90). In contrast, A − D was associated with a significant decrease in PTH levels, from 385 ± 325 ng/l to 265 ± 242 ng/l (*P* < .01) with a significant time by dialysate interaction (*P* = .02). Serum phosphate, calcium, and 25-hydroxyvitamin D levels showed no significant time-by-dialysate interaction (Supplementary Table S4).

### Safety

Adverse events were broadly comparable between dialysate periods. Four cardiovascular events occurred during A − D use, compared with five during A + D use. Infectious events were also similar, with 20 infections reported under A + D (including 15 respiratory infections) and 19 infections under A − D (including 16 respiratory infections). In the time-dependent marginal Cox proportional hazards model no statistically significant difference in mortality was observed between the two dialysate strategies. The hazard ratio for A − D compared with A + D was 0.36 (95% CI 0.10–1.36, *P* = .13) (Supplementary Fig. S1).

## DISCUSSION

We hypothesized that hydrochloric-acid-based acetate-free dialysate would favorably affect nutritional and muscle status in chronic hemodialysis patients. However, no significant differences were observed across endpoints. Although well tolerated, acetate-free dialysate showed no measurable effect on nutritional or muscle status.

A Japanese study investigated acetate-free biofiltration and its impact on nutritional and inflammatory parameters [13]. This technique uses a bicarbonate-free dialysate combined with post-dilution sodium bicarbonate reinfusion to correct metabolic acidosis. In this non-randomized study, 24 chronic hemodialysis patients were treated sequentially for 3 months with conventional hemodiafiltration using a dialysate containing 8 mmol/l acetate, followed by 3 months of acetate-free biofiltration. During the acetate-free phase, CRP and IL-6 decreased, alongside lower leptin, higher neuropeptide Y and a higher creatinine index. These findings suggested that acetate removal may attenuate inflammation and favor nutritional status. The acetate concentration in the conventional dialysate was higher than in ours, 8 versus 3 mmol/l. In addition, metabolic acidosis was less well controlled in the Japanese study, with a mean bicarbonate level of 19 mmol/l, compared with 23.6 mmol/l in our cohort. The bicarbonate concentration used during acetate-free biofiltration was also higher than in our study. Thus, the reported benefits may have been more closely related to better correction of metabolic acidosis than to acetate removal per se. Finally, the nonrandomized before–after design limits the strength of the evidence and may have introduced bias.

Two studies have compared hydrochloric-acid-based acetate-free dialysate with conventional acetate-containing dialysate and assessed inflammatory or nutritional parameters. Pizzarelli *et al*. [14] randomized 12 patients treated with online hemodiafiltration to one session with acetate-free dialysate and one with acetate-containing dialysate, containing 3 mmol/l acetate, in a crossover sequence. Plasma acetate increased significantly with acetate-containing dialysate, reaching 0.3 mmol/l at the end of the session, but CRP and IL-6 levels did not differ between treatments. However, the study was limited to single dialysis sessions, precluding assessment of nutritional status or muscle mass. Coll *et al*. [15] studied 14 chronic hemodialysis patients treated sequentially for 1 month with acetate-containing dialysate, containing 4 mmol/l acetate, followed by 1 month of acetate-free dialysate. IL-6 levels decreased modestly during the acetate-free phase, whereas CRP and serum albumin remained unchanged. TNF-α and lipopolysaccharide-binding protein levels were higher with acetate-free dialysate, suggesting a complex and not uniformly favorable inflammatory response. In our study, CRP levels did not significantly change with A − D compared with A + D.

Body weight decreased during the A − D phase, with a nonsignificant decrease in fat tissue mass. Lean tissue mass, overhydration, muscle function, and nutritional biomarkers remained stable, arguing against clinically meaningful protein-energy wasting. Bioimpedance measurement variability may explain the nonsignificant fat-mass reduction. Improved dialysis tolerance may have allowed a gradual reduction in prescribed dry weight, although unchanged overhydration provides limited support for this explanation. Exploratory analyses also suggested lower PTH with A − D. Similar findings have been reported with hydrochloric-acid-based dialysate [16] and may reflect altered calcium transfer or pH-dependent changes in ionized calcium. However, the trial was not designed to assess mineral metabolism, relevant mineral-metabolism treatments were not collected, and neither ionized calcium nor calcium balance was measured.

Our study has several limitations. First, it may have been underpowered relative to the initial sample size assumptions. The in-center hemodialysis population is characterized by multiple comorbidities and frequent intercurrent proinflammatory events, which may have masked potential dialysate-related effects. Attrition due to death, transfer to another center, or kidney transplantation also likely reduced the effective sample size over time. Second, the absence of a washout period may have resulted in residual treatment effects during the second study period, particularly for outcomes that change slowly over time. Although no significant sequence effect was detected and the analysis restricted to the first period yielded results consistent with the main analysis, neither approach can completely exclude a carryover effect. Third, Hydrochloric-acid-based dialysate provides lower net alkali delivery for a given prescribed bicarbonate concentration. Replacing 3 mmol/l acetic acid with 2 mmol/l hydrochloric acid removes 3 mmol/l of acetate-derived buffer, while reducing bicarbonate consumption during dialysate preparation by 1 mmol/l. The expected net reduction in alkali delivery is therefore 2 mmol/l, which was compensated for by increasing the prescribed dialysate bicarbonate concentration by 2 mmol/l. However, despite this adjustment, serum bicarbonate decreased by ∼1 mmol/l in our study. This may reflect imperfect agreement between conductivity-based prescription settings and the actual ionic concentrations delivered in the dialysate. Finally, muscle mass assessment by bioimpedance analysis remains debated because it may be influenced by hydration status. Although bioimpedance spectroscopy accounts for excess hydration, fluid, and electrolyte shifts related to body position and dialysis may still affect measurements. Post-dialysis assessment after a 30-min recovery period is commonly recommended, but evidence regarding its impact is inconsistent [17], and this approach is often poorly accepted by patients.

In this randomized crossover study, acetate-free dialysate was not associated with better protein–energy wasting parameters, muscle mass, or inflammatory markers than conventional acetate-containing dialysate. Both dialysate compositions were well tolerated, with similar safety profiles. In a contemporary hemodialysis setting with low acetate exposure and adequate metabolic acidosis control, acetate removal alone does not appear to provide nutritional benefit. Larger randomized trials are needed to determine whether acetate-free dialysate may improve long-term survival.

## Supplementary Material

sfag323_Supplemental_File

## Data Availability

Study data are available from the corresponding author upon reasonable request, in accordance with institutional and ethical guidelines.
