# Supplementary material for: Acetate-free dialysate, protein-energy wasting, and muscle outcomes in maintenance hemodialysis: a randomized-controlled trial
Source: Clin Kidney J. 2026 Sep 22;19(10):sfag323. doi: 10.1093/ckj/sfag323 (PMC13639479; doi:10.1093/ckj/sfag323)
Supplement: Supplementary file 1 [file sfag323_supplemental_file.docx]

**Supplementary Data**

**Supplementary Table 1. Number of patients with available data for each outcome according to treatment sequence, study period, dialysate, and assessment time**

| **Outcome** | **Sequence A+D then A-D (n = 30)** | | | | **Sequence A-D then A+D (n = 28)** | | | |
| --- | --- | --- | --- | --- | --- | --- | --- | --- |
|  | **Period 1: A+D** | | **Period 2: A-D** | | **Period 1: A-D** | | **Period 2: A+D** | |
|  | **M0** | **M6** | **M0** | **M6** | **M0** | **M6** | **M0** | **M6** |
| Albumin | 30 | 26 | 26 | 21 | 28 | 25 | 25 | 22 |
| Calcium | 29 | 25 | 25 | 21 | 28 | 25 | 25 | 22 |
| CRP | 30 | 26 | 26 | 21 | 28 | 25 | 25 | 22 |
| FTI | 28 | 24 | 24 | 20 | 28 | 24 | 24 | 20 |
| FTM | 28 | 24 | 24 | 20 | 28 | 24 | 24 | 20 |
| LTI | 28 | 24 | 24 | 20 | 28 | 24 | 24 | 20 |
| LTM | 28 | 24 | 24 | 20 | 28 | 24 | 24 | 20 |
| Maximal gripping force | 30 | 26 | 26 | 21 | 28 | 25 | 25 | 22 |
| Average gripping force | 30 | 26 | 26 | 21 | 28 | 25 | 25 | 22 |
| Overhydration | 28 | 24 | 24 | 20 | 28 | 24 | 24 | 20 |
| 25-OH vitamin D | 29 | 26 | 26 | 21 | 28 | 25 | 25 | 22 |
| Phosphate | 30 | 26 | 26 | 21 | 28 | 24 | 24 | 22 |
| Prealbumin | 30 | 26 | 26 | 21 | 28 | 25 | 25 | 22 |
| PTH | 29 | 26 | 26 | 21 | 28 | 25 | 25 | 22 |
| SARC-F score | 30 | 26 | 26 | 21 | 28 | 25 | 25 | 22 |
| 4-m gait speed | 30 | 26 | 26 | 21 | 25 | 24 | 24 | 19 |
| Weight | 30 | 26 | 26 | 21 | 28 | 25 | 25 | 22 |

Data are the number of patients with an available measurement for each outcome. M0 and M6 correspond to the beginning and end of each 6-month treatment period, respectively. Differences in sample size across outcomes reflect missing measurements. A-D, acetate-free dialysate; A+D, acetate-containing dialysate. CRP, C-reactive protein; ECW, extracellular water; FTI, fat tissue index; FTM, fat tissue mass; LTI, lean tissue index; LTM, lean tissue mass; PTH, parathyroid hormone.

**Supplementary Table 2. Endpoints related to protein-energy wasting and muscle outcomes (per-protocol analysis)**

|  | **GrouP A-D** | | | **Group A+D** | | |  |  |  |
| --- | --- | --- | --- | --- | --- | --- | --- | --- | --- |
|  | **M0**  **n=39** | **M6**  **n=39** | p | **M0**  **n=39** | **M6**  **n=39** | **p** | **p^1^** | **p^2^** | **p^3^** |
| Primary endpoint |  |  |  |  |  |  |  |  |  |
| Prealbumin (mg/L) | 292.3 ± 85.9 | 281.8 ± 71.0 | 0.36 | 287.2 ± 69.0 | 283.6 ± 70.4 | 0.70 | 0.60 | 0.80 | 0.30 |
| Secondary endpoints |  |  |  |  |  |  |  |  |  |
| Weight (kg) | 74.4 ± 18.2 | 73.3 ± 18.0 | 0.01 | 73.7 ± 18.1 | 73.9 ± 18.3 | 0.63 | 0.02 | 0.66 | 0.09 |
| LTI (kg/m^2^) | 11.9 ± 2.9 | 11.7 ± 2.6 | 0.16 | 11.6 ± 2.7 | 11.6 ± 3.0 | 0.81 | 0.77 | 0.79 | 0.75 |
| LTM (kg) | 34.4 ± 9.5 | 33.7 ± 8.7 | 0.13 | 33.4 ± 9.1 | 33.3 ± 9.7 | 0.82 | 0.78 | 0.48 | 0.73 |
| FTI (kg/m^2^) | 14.1 ± 7.3 | 13.8 ± 6.7 | 0.45 | 14.0 ± 7.0 | 14.5 ± 7.7 | 0.96 | 0.56 | 0.71 | 0.72 |
| FTM (kg) | 29.4 ±14.9 | 29.7  ± 14.3 | 0.42 | 29.8 ± 14.8 | 30.0 ± 15.6 | 0.96 | 0.61 | 0.75 | 0.62 |
| OH (L) | 0.98 ± 1.64 | 1.02 ± 1.58 | 0.88 | 0.82 ± 1.60 | 0.59 ± 1.61 | 0.46 | 0.64 | 0.63 | 0.67 |
| Average gripping force (kg) | 25.6 ± 9.0 | 26.1 ± 9.8 | 0.63 | 25.8 ± 9.7 | 26.0 ± 10.3 | 0.79 | 0.78 | 0.45 | 0.57 |
| Maximal gripping force (kg) | 27.2 ± 8.9 | 27.5 ± 10.0 | 0.78 | 27.1 ± 10.0 | 27.4 ± 10.3 | 0.98 | 0.96 | 0.46 | 0.62 |
| SARC-F | 2.1 ± 2.2 | 2.3 ± 2.5 | 0.58 | 2.5 ± 2.4 | 2.6 ± 2.6 | 0.83 | 0.72 | 0.35 | 0.71 |
| 4m gait speed (m/s) | 0.84 ± 0.27 | 0.88 ± 0.24 | 0.36 | 0.83 ± 0.25 | 0.88 ± 0.30 | 0.053 | 0.41 | 0.83 | 0.03 |
| Albumin (g/L) | 36.4 ± 4.2 | 36.1 ± 3.4 | 0.94 | 36.0 ± 3.5 | 35.8 ± 2.9 | 0.80 | 0.91 | 0.94 | 0.72 |
| CRP (mg/L) | 10.4 ± 12.3 | 15.0 ± 25.1 | 0.30 | 15.0 ± 23.2 | 11.3 ± 13.2 | 0.35 | 0.09 | 0.18 | 0.88 |

Continuous variables are expressed as mean ± standard deviation. A-D, acetate free dialysate ; A+D, acetate containing dialysate ; CRP, C-reactive protein ; FTI, fat tissue index ; FTM, fat tissue mass, LTI, lean tissu index ; LTM, lean tissue mass ; OH, overhydratation. p : p value for time effect adjusted for period with each dialysate. p^1^ : p value for the dialysate-by-time interaction . p^2^ : p value for sequence effect. p^3^ : p value for period effect.

**Supplementary Table 3. Analysis by study period, reporting p values for the dialysate-by-time interaction.**

|  | **p Period 1**  **(Intent-to treat analysis)** | **p Period 1**  **(Per-Protocol analysis)** |
| --- | --- | --- |
| Primary endpoint |  |  |
| Prealbumin (mg/L) | 0.61 | 0.81 |
| Secondary endpoints |  |  |
| Weight (kg) | 0.06 | 0.11 |
| LTI (kg/m^2^) | 0.88 | 0.11 |
| LTM (kg) | 0.85 | 0.10 |
| FTI (kg/m^2^) | 0.73 | 0.18 |
| FTM (kg) | 0.72 | 0.20 |
| OH (L) | 0.89 | 0.37 |
| Average gripping force (kg) | 0.95 | 0.96 |
| Maximal gripping force (kg) | 0.93 | 0.86 |
| SARC-F | 0.07 | 0.24 |
| 4m gait speed (m/s) | 0.46 | 0.36 |
| Albumin (g/L) | 0.87 | 0.67 |
| CRP (mg/L) | 0.12 | 0.31 |

**Supplementary Table 4. Effect of dialysate on mineral and bone disorder parameters**

|  | **GrouP A-D** | | | **Group A+D** | | |  |  |  |
| --- | --- | --- | --- | --- | --- | --- | --- | --- | --- |
|  | **M0**  **n=54** | **M6**  **n=46** | p | **M0**  **n=55** | **M6**  **n=48** | **p** | **p^1^** | **p^2^** | **p^3^** |
| PTH (ng/mL) | 385 ± 325 | 265 ± 242 | <0.01 | 341 ± 293 | 346 ± 276 | 0.90 | 0.02 | 0.28 | 0.04 |
| 25-OH D3 | 36.0 ± 12.1 | 38.6 ±13.7 | 0.26 | 34.1 ± 11.9 | 36.3 ±12.9 | 0.23 | 0.77 | 0.31 | 0.21 |
| Calcium | 2.23 ± 0.14 | 2.26 ± 0.16 | 0.26 | 2.21 ± 0.17 | 2.26 ± 0.15 | <0.01 | 0.27 | 0.61 | 0.51 |
| Phosphorus | 1.27 ±0.41 | 1.16 ± 0.44 | 0.17 | 1.22 ± 0.48 | 1.25 ± 0.50 | 0.61 | 0.17 | 0.64 | 0.19 |

Continuous variables are expressed as mean ± standard deviation. A-D, acetate free dialysate ; A+D, acetate containing dialysate ; 25 OH D3, 25-hydroxyvitamin D3 ; PTH, parathyroid hormone. p^1^ : p value for effect of time x dialysate interaction . p^2^ : p value for effect of timexdialysate interaction only period 1. p^3^ : p value for effect of timexdialysate interaction only period 2

**Supplementary Figure 1. Adjusted survival according to dialysate exposure.**
Survival curves are shown according to dialysate exposure, comparing acetate-free dialysate (A−D) with acetate-containing dialysate (A+D). Survival was analyzed using a marginal Cox proportional hazards model with dialysate exposure treated as a time-varying covariate. Within-patient correlation related to the crossover design was accounted for using clustered robust standard errors, and the model was adjusted for age, sex, and Charlson comorbidity index.
